# The genome sequence of *Gari tellinella *(Lamarck, 1818), a sunset clam

**DOI:** 10.12688/wellcomeopenres.17805.1

**Published:** 2022-03-29

**Authors:** Anna Holmes, Teresa Darbyshire, Mitchell Brennan, Sean McTierney, Allison Small, Marine Biological Association Genome Acquisition Lab, Darwin Tree of Life Barcoding collective, Wellcome Sanger Institute Tree of Life programme, Wellcome Sanger Institute Scientific Operations: DNA Pipelines collective, Tree of Life Core Informatics collective, Darwin Tree of Life Consortium

**Affiliations:** 1 National Museum of Wales, Cardiff, UK; 2 Marine Biological Association, Plymouth, Devon, UK

**Keywords:** Gari tellinella, clam, genome sequence, chromosomal, Bivalvia

## Abstract

We present a genome assembly from an individual *Gari tellinella* (Mollusca; Bivalvia; Cardiida; Psammobiidae). The genome sequence is 1,598 megabases in span. The majority of the assembly (99.85%) is scaffolded into 19 chromosomal pseudomolecules. The complete mitochondrial genome was also assembled and is 18.5 kilobases in length.

## Species taxonomy

Eukaryota; Metazoa; Spiralia; Lophotrochozoa; Mollusca; Bivalvia; Autobranchia; Heteroconchia; Euheterodonta; Imparidentia; Neoheterodontei; Cardiida; Tellinoidea; Psammobiidae; Gari; *Gari tellinella* (Lamarck, 1818) (NCBI:txid2589376).

## Background

*Gari tellinella* belongs to a group of bivalves known as the sunset clams (Psammobiidae). This small (up to 26 mm), thin-shelled, often colourful bivalve is found in silty coarse sands and shell gravel from the intertidal to shelf depths around most of the UK, except the southern North Sea. Its range extends from northern France through to Norway and Sweden, but sporadic records are also noted from northwestern and southern Spain and a few from the Mediterranean.

Flattened, elongated oval in outline, externally *G. tellinella* is sculpted by concentric lines and clear growth stops and bears a smooth outer shell margin. Cream, pale brown, orange, purple or white in colour, it frequently has umbonal rays of orange, red or white that extend to the outer margin. Internally, *G. tellinella* can be orange, mustard yellow or white, again, with umbonal rays visible as white or reddish orange. The pallial sinus that runs between the two adductor muscles is half the length of the shell and narrowly curved; the bottom half is confluent with the pallial line.

*Gari tellinella* is most similar to *Gari depressa*, but is a third of the size and has a narrowly rounded posterior margin, which compares to the almost truncate posterior of *G. depressa*. There is also no posterior gape between the valves in *G. tellinella*, but a small gape in *G. depressa*.

## Genome sequence report

**Figure 1.**
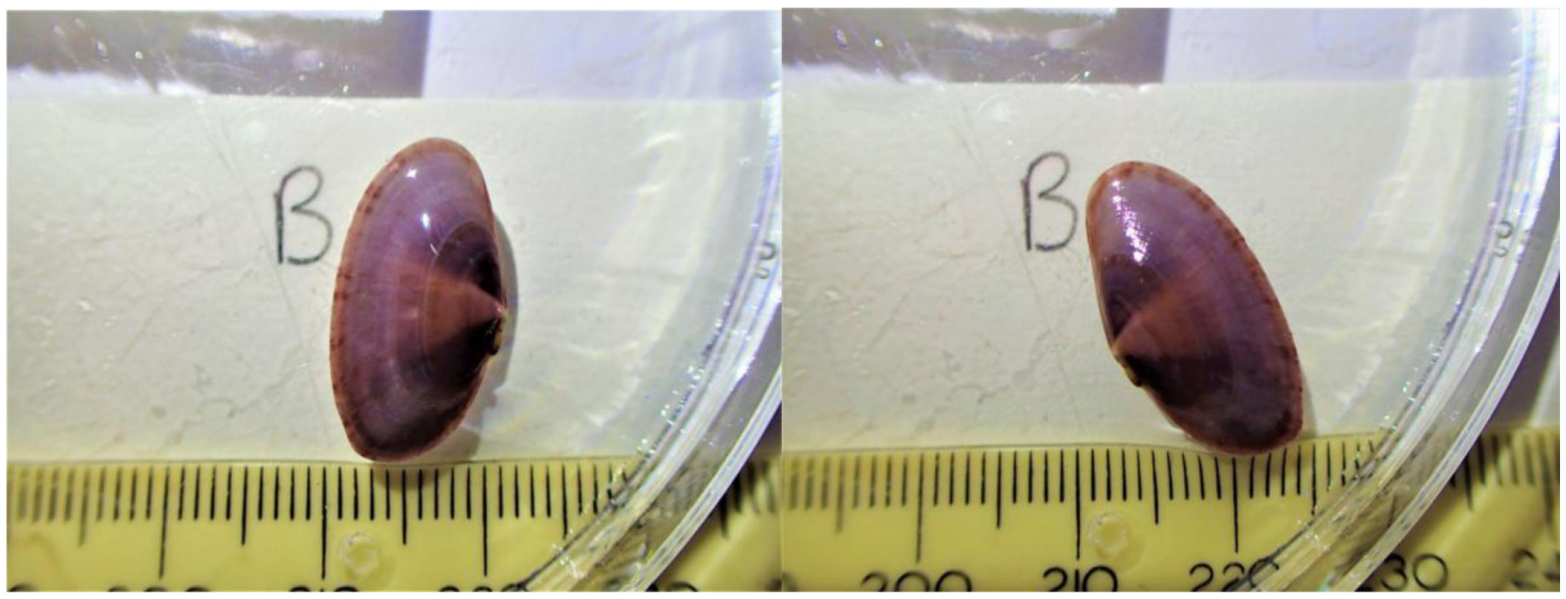
Images of the *Gari tellinella* specimen taken during preservation and processing.

The genome was sequenced from a single *G. tellinella* collected from Jennycliff Bay, Plymouth Sound, Plymouth, UK ( Figure 1). A total of 34-fold coverage in Pacific Biosciences single-molecule HiFi long reads and 25-fold coverage in 10X Genomics read clouds were generated. Primary assembly contigs were scaffolded with chromosome conformation Hi-C data. Manual assembly curation corrected 116 missing/misjoins and removed 11 haplotypic duplications, reducing the assembly size by 0.40% and the scaffold number by 36.72%, and increasing the scaffold N50 by 1.61%.

**Figure 2.**
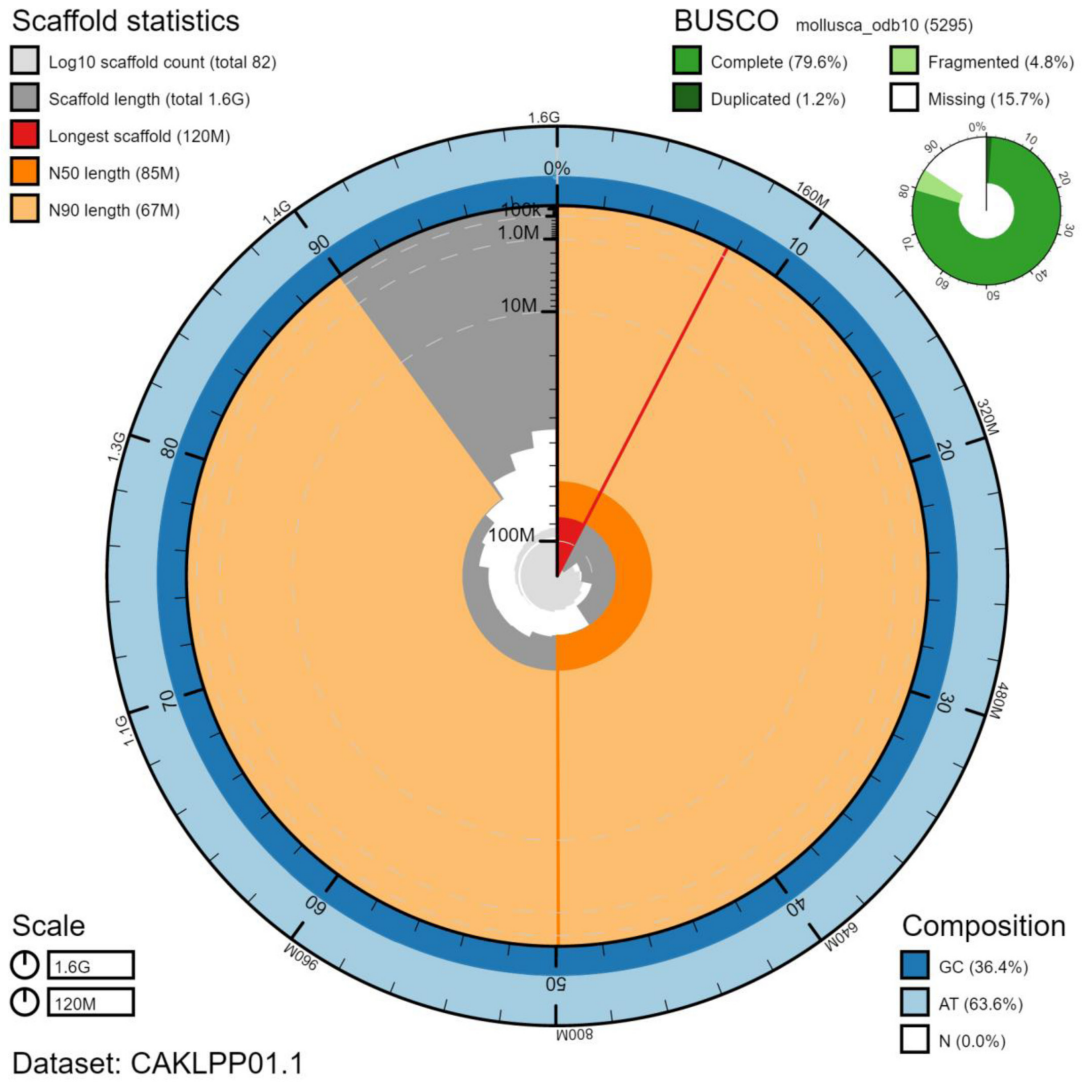
Genome assembly of *Gari tellinella*, xbGarTell2.1: metrics. The BlobToolKit Snailplot shows N50 metrics and BUSCO gene completeness. The main plot is divided into 1,000 size-ordered bins around the circumference with each bin representing 0.1% of the 1,597,633,241 bp assembly. The distribution of chromosome lengths is shown in dark grey with the plot radius scaled to the longest chromosome present in the assembly (121,778,318 bp, shown in red). Orange and pale-orange arcs show the N50 and N90 chromosome lengths (85,279,272 and 67,439,623 bp), respectively. The pale grey spiral shows the cumulative chromosome count on a log scale with white scale lines showing successive orders of magnitude. The blue and pale-blue area around the outside of the plot shows the distribution of GC, AT and N percentages in the same bins as the inner plot. A summary of complete, fragmented, duplicated and missing BUSCO genes in the mollusca_odb10 set is shown in the top right. An interactive version of this figure is available at https://blobtoolkit.genomehubs.org/view/xbGarTell2.1/dataset/CAKLPP01.1/snail.

The final assembly has a total length of 1,598 Mb in 81 sequence scaffolds with a scaffold N50 of 85.3 Mb ( Table 1). The majority, 99.85%, of the assembly sequence was assigned to 19 chromosomal-level scaffolds, representing 19 autosomes (numbered by sequence length) ( Figure 2– Figure 5; Table 2). Chromosome 9 consists of a mosaic of haplotypes that is arranged into one of two valid structural possibilities.

**Table 1.** Genome data for *Gari tellinella*, xbGarTell2.1.

*Project accession data*	
Assembly identifier	xbGarTell2.1
Species	*Gari tellinella*
Specimen	xbGarTell2
NCBI taxonomy ID	2589376
BioProject	PRJEB48048
BioSample ID	SAMEA8724799
Isolate information	xbGarTell2, other somatic tissue
*Raw data accessions*	
PacificBiosciences SEQUEL II	ERR7123968-ERR7123970
10X Genomics Illumina	ERR7113546-ERR7113549
Hi-C Illumina	ERR7113550
*Genome assembly*	
Assembly accession	GCA_922989275.1
*Accession of alternate* *haplotype*	GCA_922984925.1
Span (Mb)	1,598
Number of contigs	260
Contig N50 length (Mb)	19.2
Number of scaffolds	81
Scaffold N50 length (Mb)	85.3
Longest scaffold (Mb)	122
BUSCO * genome score	C:79.6%[S:78.4%,D:1.2%],F:4.8%, M:15.7%,n:5295

*BUSCO scores based on the metazoa_odb10 BUSCO set using v5.1.2. C= complete [S= single copy, D=duplicated], F=fragmented, M=missing, n=number of orthologues in comparison. A full set of BUSCO scores is available at https://blobtoolkit.genomehubs.org/view/xbGarTell2.1/dataset/CAKLPP01.1/busco.

**Figure 3.**
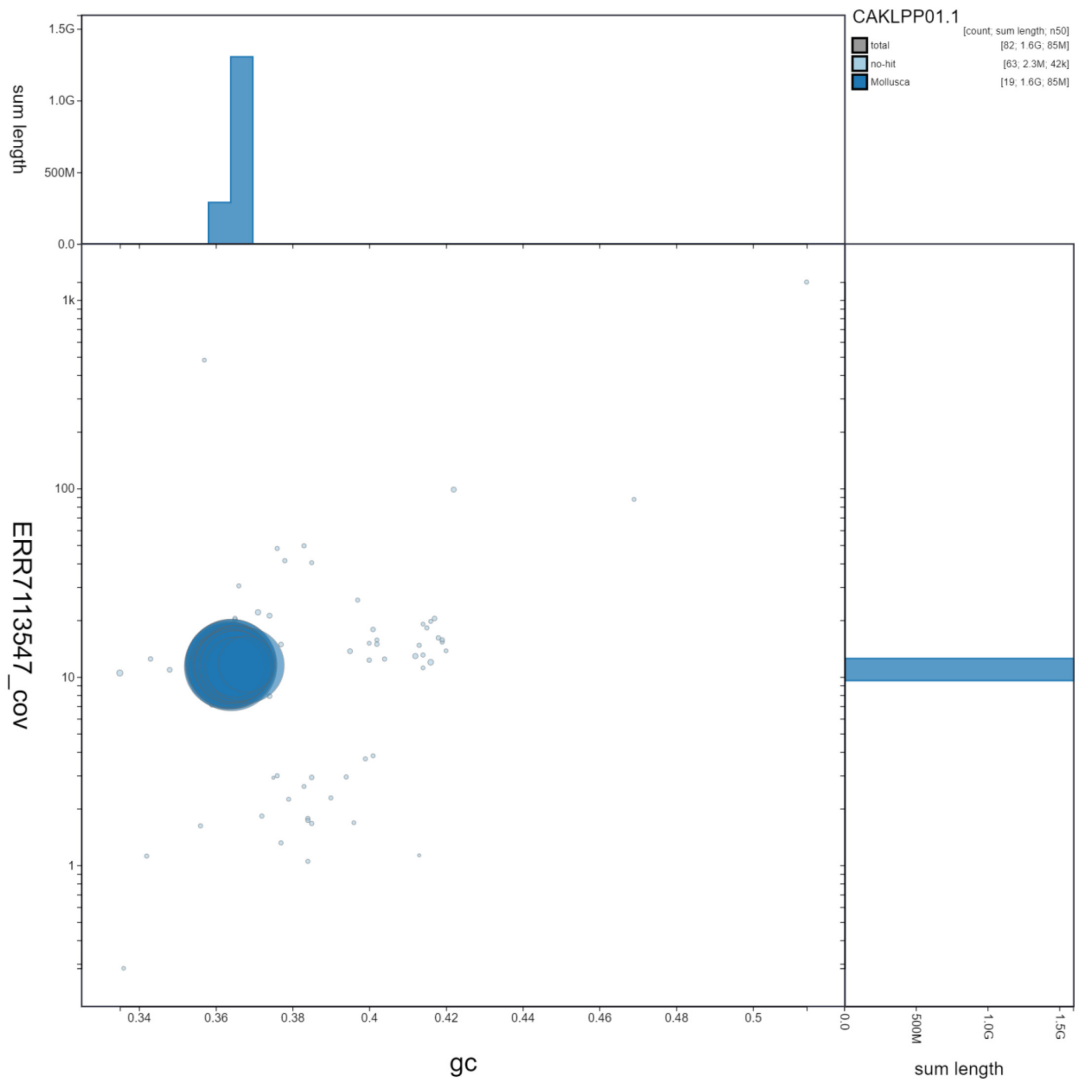
Genome assembly of *Gari tellinella*, xbGarTell2.1: GC coverage. BlobToolKit GC-coverage plot. Scaffolds are coloured by phylum. Circles are sized in proportion to scaffold length. Histograms show the distribution of scaffold length sum along each axis. An interactive version of this figure is available at https://blobtoolkit.genomehubs.org/view/xbGarTell2.1/dataset/CAKLPP01.1/blob.

**Figure 4.**
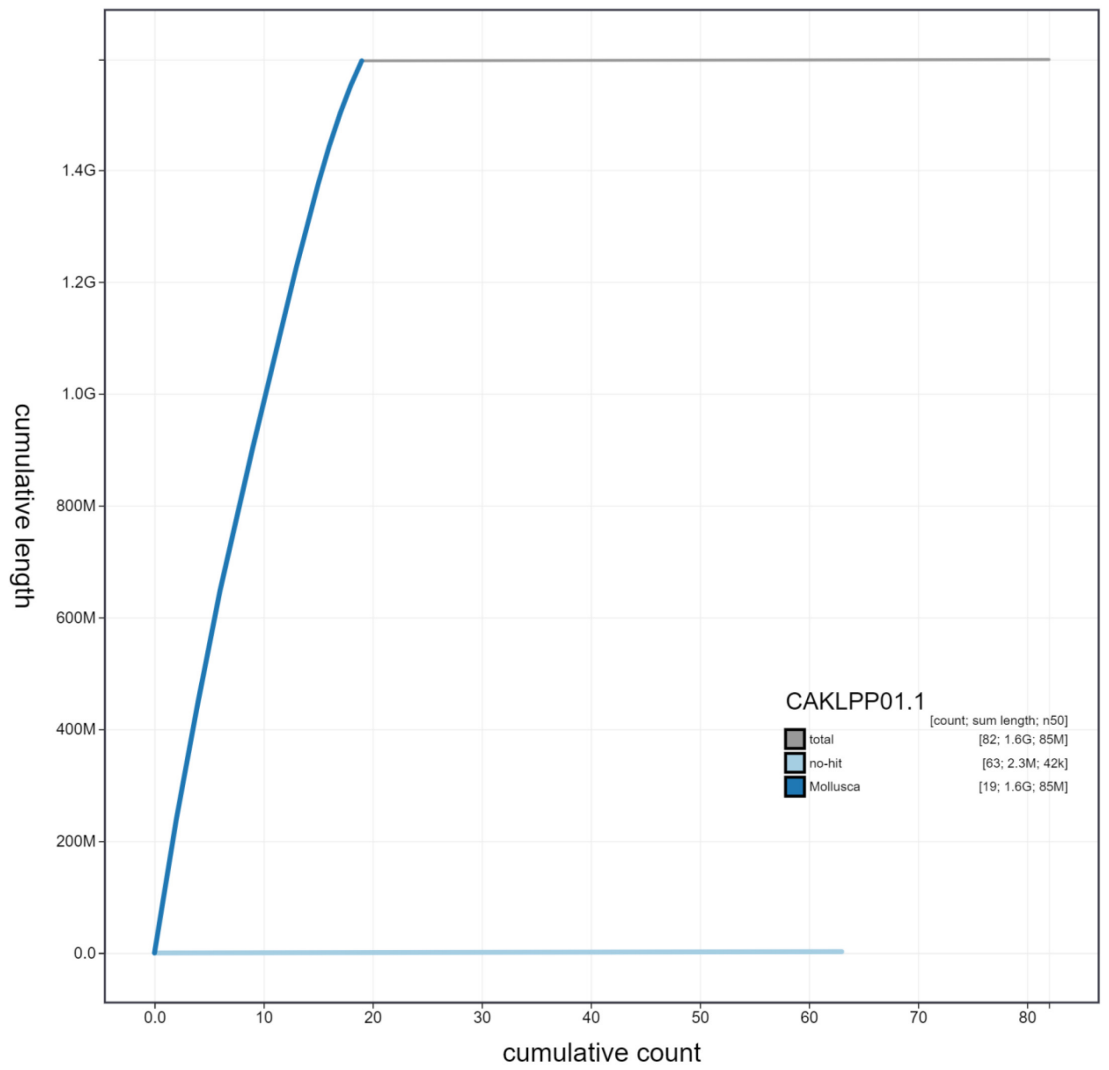
Genome assembly of *Gari tellinella*, xbGarTell2.1: cumulative sequence. BlobToolKit cumulative sequence plot. The grey line shows cumulative length for all scaffolds. Coloured lines show cumulative lengths of scaffolds assigned to each phylum using the buscogenes taxrule. An interactive version of this figure is available at https://blobtoolkit.genomehubs.org/view/xbGarTell2.1/dataset/CAKLPP01.1/cumulative.

**Figure 5.**
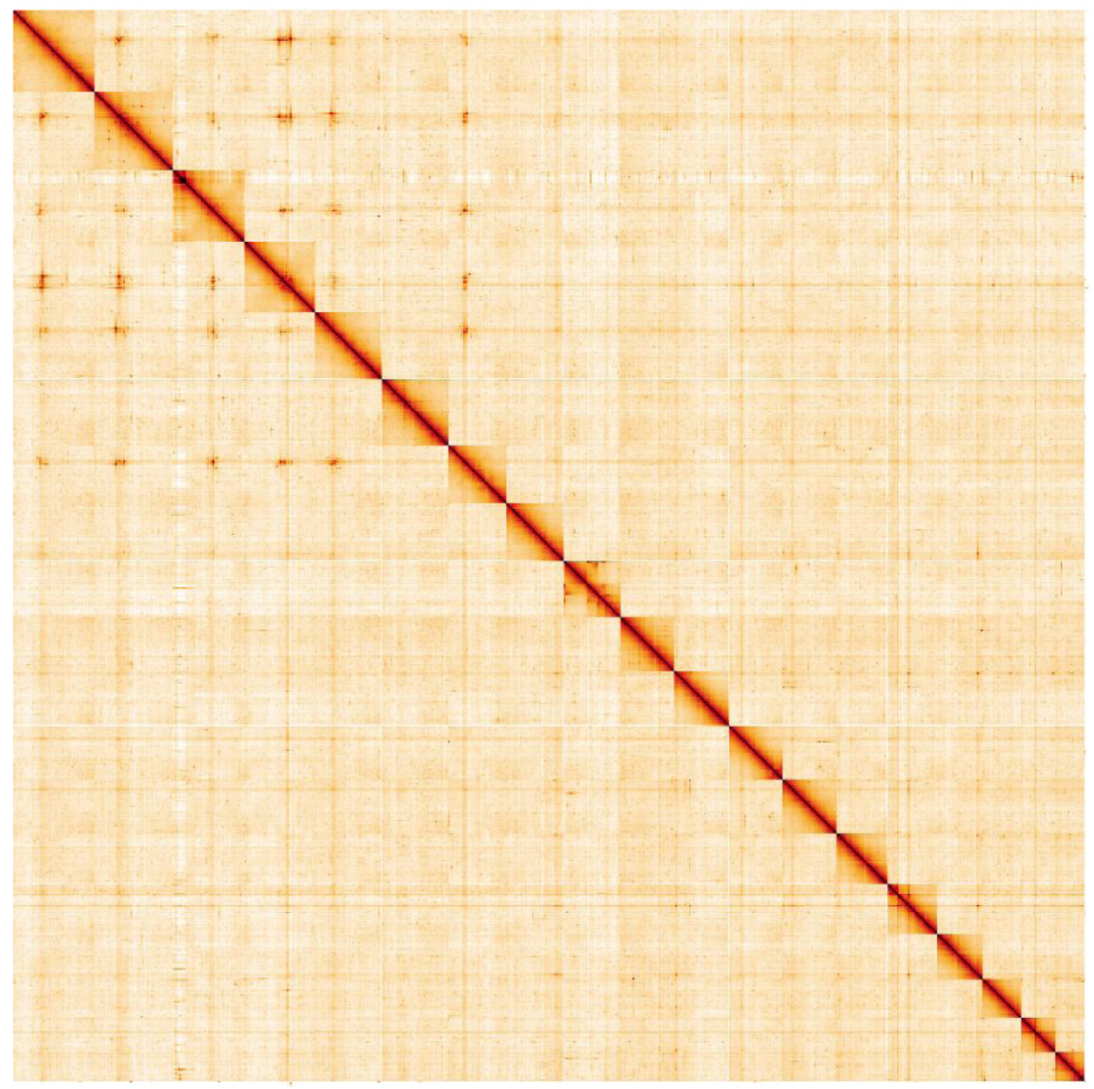
Genome assembly of *Gari tellinella*, xbGarTell2.1: Hi-C contact map. Hi-C contact map of the xbGarTell2.1 assembly, visualised in HiGlass. Chromosomes are arranged in size order from left to right and top to bottom. The interactive Hi-C map can be viewed here.

**Table 2.** Chromosomal pseudomolecules in the genome assembly of *Gari tellinella*, xbGarTell2.1.

INSDC accession	Chromosome	Size (Mb)	GC%
OV277856.1	1	121.78	36.4
OV277857.1	2	116.39	36.4
OV277858.1	3	106.25	36.3
OV277859.1	4	105.67	36.4
OV277860.1	5	99.00	36.4
OV277861.1	6	98.98	36.3
OV277862.1	7	85.92	36.4
OV277863.1	8	85.28	36.5
OV277864.1	9	84.53	36.3
OV277865.1	10	81.19	36.8
OV277866.1	11	80.75	36.4
OV277867.1	12	80.47	36.4
OV277868.1	13	80.23	36.5
OV277869.1	14	75.02	36.4
OV277870.1	15	73.21	36.5
OV277871.1	16	67.44	36.5
OV277872.1	17	58.22	36.6
OV277873.1	18	50.86	36.5
OV277874.1	19	44.11	36.8
OV277875.1	MT	0.02	35.8
-	Unplaced	2.31	38.7

The assembly has a BUSCO v5.1.2 ( Manni *et al*., 2021) completeness of 79.6% (single 78.4%, duplicated 1.2%) using the mollusca_odb10 reference set (n=5294). However, we believe that this relatively low BUSCO score is a result of limitations with the current mollusca_odb10 geneset. Using the metazoa_odb10 reference set (n=954), the assembly has a completeness of 94.9% (single 94.2%, duplicated 0.7%), which we believe is evidence of high completeness. While not fully phased, the assembly deposited is of one haplotype. Contigs corresponding to the second haplotype have also been deposited.

## Methods

### Sample acquisition and DNA extraction

A single *G. tellinella* specimen (xbGarTell2) was collected by boat from sand in Jennycliff Bay, Plymouth Sound, Plymouth, UK (latitude 50.3394, longitude -4.1311) by Teresa Derbyshire (National Museum of Wales), Mitchell Brennan, Sean McTierney and Allison Small (Marine Biological Association). The specimen was identified by Anna Holmes (National Museum of Wales) and snap-frozen in liquid nitrogen.

DNA was extracted at the Tree of Life laboratory, Wellcome Sanger Institute. The xbGarTell2 sample was weighed and dissected on dry ice with tissue set aside for Hi-C and RNA sequencing. Tissue was disrupted using a Nippi Powermasher fitted with a BioMasher pestle. Fragment size analysis of 0.01–0.5 ng of DNA was then performed using an Agilent FemtoPulse. High molecular weight (HMW) DNA was extracted using the Qiagen MagAttract HMW DNA extraction kit. Low molecular weight DNA was removed from a 200-ng aliquot of extracted DNA using 0.8X AMpure XP purification kit prior to 10X Chromium sequencing; a minimum of 50 ng DNA was submitted for 10X sequencing. HMW DNA was sheared into an average fragment size between 12–20 kb in a Megaruptor 3 system with speed setting 30. Sheared DNA was purified by solid-phase reversible immobilisation using AMPure PB beads with a 1.8X ratio of beads to sample to remove the shorter fragments and concentrate the DNA sample. The concentration of the sheared and purified DNA was assessed using a Nanodrop spectrophotometer and Qubit Fluorometer and Qubit dsDNA High Sensitivity Assay kit. Fragment size distribution was evaluated by running the sample on the FemtoPulse system.

### Sequencing

Pacific Biosciences HiFi circular consensus and 10X Genomics Chromium read cloud sequencing libraries were constructed according to the manufacturers’ instructions. Sequencing was performed by the Scientific Operations core at the Wellcome Sanger Institute on Pacific Biosciences SEQUEL II (HiFi) and Illumina NovaSeq 6000 (10X) instruments. Hi-C data were generated in the Tree of Life laboratory from remaining tissue of xbGarTell2 using the Arima v2 kit and sequenced on a NovaSeq 6000 instrument.

### Genome assembly

Assembly was carried out with Hifiasm ( Cheng *et al*., 2021); haplotypic duplication was identified and removed with purge_dups ( Guan *et al*., 2020). One round of polishing was performed by aligning 10X Genomics read data to the assembly with longranger align, calling variants with freebayes ( Garrison & Marth, 2012). The assembly was then scaffolded with Hi-C data ( Rao *et al*., 2014) using yahs. The assembly was checked for contamination and corrected using gEVAL ( Chow *et al*., 2016) as described previously ( Howe *et al*., 2021). Manual curation ( Howe *et al*., 2021) was performed using gEVAL, HiGlass ( Kerpedjiev *et al*., 2018) and Pretext. The mitochondrial genome was assembled using MitoHiFi ( Uliano-Silva *et al*., 2021), which performs annotation using MitoFinder ( Allio *et al*., 2020). The genome was analysed and BUSCO scores generated within the BlobToolKit environment ( Challis *et al*., 2020). Table 3 contains a list of all software tool versions used, where appropriate.

**Table 3.** Software tools used.

Software tool	Version	Source
Hifiasm	0.15.3	Cheng *et al*., 2021
purge_dups	1.2.3	Guan *et al*., 2020
yahs	1.0	https://github.com/c-zhou/yahs
longranger align	2.2.2	https://support.10xgenomics.com/ genome-exome/software/pipelines/latest/ advanced/other-pipelines
freebayes	1.3.1-17-gaa2ace8	Garrison & Marth, 2012
MitoHiFi	2.0	Uliano-Silva *et al*., 2021
gEVAL	N/A	Chow *et al*., 2016
HiGlass	1.11.6	Kerpedjiev *et al*., 2018
PretextView	0.2.x	https://github.com/wtsi-hpag/PretextView
BlobToolKit	3.0.5	Challis *et al*., 2020

### Ethics/compliance issues

The materials that have contributed to this genome note have been supplied by a Darwin Tree of Life Partner. The submission of materials by a Darwin Tree of Life Partner is subject to the Darwin Tree of Life Project Sampling Code of Practice. By agreeing with and signing up to the Sampling Code of Practice, the Darwin Tree of Life Partner agrees they will meet the legal and ethical requirements and standards set out within this document in respect of all samples acquired for, and supplied to, the Darwin Tree of Life Project. Each transfer of samples is further undertaken according to a Research Collaboration Agreement or Material Transfer Agreement entered into by the Darwin Tree of Life Partner, Genome Research Limited (operating as the Wellcome Sanger Institute), and in some circumstances other Darwin Tree of Life collaborators.

## Data availability

European Nucleotide Archive: Gari tellinella. Accession number PRJEB48048; https://identifiers.org/ena.embl/PRJEB48048.

The genome sequence is released openly for reuse. The *G. tellinella* genome sequencing initiative is part of the Darwin Tree of Life (DToL) project. All raw sequence data and the assembly have been deposited in INSDC databases. The genome will be annotated and presented through the Ensembl pipeline at the European Bioinformatics Institute. Raw data and assembly accession identifiers are reported in Table 1.

## Author information

Members of the Marine Biological Association Genome Acquisition Lab are listed here: https://doi.org./10.5281/zenodo.5913830.

Members of the Darwin Tree of Life Barcoding collective are listed here: https://doi.org/10.5281/zenodo.5744972.

Members of the Wellcome Sanger Institute Tree of Life programme are listed here: https://doi.org/10.5281/zenodo.6125027.

Members of Wellcome Sanger Institute Scientific Operations: DNA Pipelines collective are listed here: https://doi.org/10.5281/zenodo.5746904.

Members of the Tree of Life Core Informatics collective are listed here: https://doi.org/10.5281/zenodo.6125046.

Members of the Darwin Tree of Life Consortium are listed here: https://doi.org/10.5281/zenodo.5638618.
