# Supplementary material for: The genome sequence of Gari tellinella (Lamarck, 1818), a sunset clam
Source: Wellcome Open Res. 2022 Mar 29;7:116. [Version 1] doi: 10.12688/wellcomeopenres.17805.1 (PMC10685066; doi:10.12688/wellcomeopenres.17805.1)
Supplement: Peer review history

# Reviewer report: Reviewer response for version 1

**DOI:** 10.21956/wellcomeopenres.19711.r54354

**Published:** 2023-11-28

**Authors:** Chase Smith

**Affiliations:** 1 University of Texas, Austin, USA

This study will be integral toward further improving bivalve genomic resources and provides a high quality genome for *Gari tellinella*. I think the authors have done a nice job presenting the study and I only have a few minor questions/suggestions before indexing.

- In Figure 2, you present the molluscan lineage BUSCO report. I agree this has issues since it is extremely limited. I would recommend replacing it with the metazoan report since it is probably a better evaluation of genome completeness. 
- In the methods under sample acquisition: "Hi-C and RNA sequencing". Assuming you mean DNA?
- The contact map is fantastic. Many researchers (including myself) have had extensive difficulties with generating libraries for chromatin sequencing (e.g., HiC, OmniC). If there were any specifics that were done to ensure proper digestion and library preparation it would be useful to add them to the Methods. 
- Any reason you did not provide an annotation for the genome? I do not think it is a necessity given the quality of this assembly but would greatly increase the use of the data. Although there is no RNAseq generated in this study available, proteins from recent bivalve genome assemblies could be used as a training dataset. 

Chase Smith

Are sufficient details of methods and materials provided to allow replication by others?

Yes

Is the rationale for creating the dataset(s) clearly described?

Yes

Are the datasets clearly presented in a useable and accessible format?

Yes

Are the protocols appropriate and is the work technically sound?

Partly

Reviewer Expertise:

Bivalve genomics

I confirm that I have read this submission and believe that I have an appropriate level of expertise to confirm that it is of an acceptable scientific standard.

# Reviewer report: Reviewer response for version 1

**DOI:** 10.21956/wellcomeopenres.19711.r57017

**Published:** 2023-07-18

**Authors:** Cristian Gallardo-Escárate

**Affiliations:** 1 University of Concepción, Concepción, Chile

The reported genome resource has the quality to be accepted in WOR. All methods and analysis conducted are well addressed and reflect the state of the art to produce a genome assembly at chromosome level. With respect to the BUSCO score, there is a limitation with the current version for mollusca_odb10 genset. Thus, I totally expected the completeness reported.

Are sufficient details of methods and materials provided to allow replication by others?

Yes

Is the rationale for creating the dataset(s) clearly described?

Yes

Are the datasets clearly presented in a useable and accessible format?

Yes

Are the protocols appropriate and is the work technically sound?

Yes

Reviewer Expertise:

Marine genomics and molecular ecology

I confirm that I have read this submission and believe that I have an appropriate level of expertise to confirm that it is of an acceptable scientific standard.

# Reviewer report: Reviewer response for version 1

**DOI:** 10.21956/wellcomeopenres.19711.r57012

**Published:** 2023-06-02

**Authors:** David Osca

**Affiliations:** 1 Instituto Universitario de Estudios Ambientales y Recursos Naturales. Faculty of Marine Sciences. Department of Biology, University of Las Palmas de Gran Canaria, Las Palmas de Gran Canaria, Canary Islands, Spain

First and foremost, I would like to extend my congratulations to the authors for their commendable efforts in working with genomes and their significant contribution through the publication of a new mollusk genome. However, I would like to offer some suggestions for improving the article, as I believe they are necessary:

**i)** In the "Background" section, it would be beneficial to include citations to support the information provided about the species and its distribution. This would help clarify if the descriptions and data presented have been obtained from previous publications.

**ii)** In the "Genome Sequence Report" and "Methods" sections, while the specific organism that has been sequenced is described, the article does not mention the sex of the individual. Although this information is typically not crucial, in the case of Bivalvia, where doubly uniparental inheritance (DUI) occurs, it is important to indicate whether the individual studied was male or female. This is particularly relevant due Psammobiidae sister groups show DUI (Smith et al., 2023) ^1^. Including this information would help avoid potential conflicts in the interpretation of the obtained data.

**iii)** It is essential to provide the voucher code assigned to the specimen studied, as well as any relevant information regarding the DNA or leftover tissue associated with the study.

**iv) **Consider using a black background, including a scale, and ensuring the correct orientation of the bivalve is depicted. These modifications will enhance the clarity and professionalism of the figure 1.

**v)** The article explains the number of chromosomes and autosomes, but there is no mention of the annotation of the mitogenome, such as the number of genes or a figure depicting their arrangement. It would be helpful to include this information to provide a comprehensive understanding of the genome.

**vi)** Although the size of the mitogenome is mentioned in the abstract, it is not explicitly stated in the main text of the article. While Table 2 makes a vague reference to the data (0.02 megabases), the abstract states a more specific size (18.5 kilobases). To ensure consistency and clarity, it is advisable to include the precise size of the mitogenome within the main body of the article.

 In conclusion, I would like to once again commend the authors for their remarkable work and valuable contribution to the field. By implementing these suggested improvements, the article will become more comprehensive.

Are sufficient details of methods and materials provided to allow replication by others?

Yes

Is the rationale for creating the dataset(s) clearly described?

Yes

Are the datasets clearly presented in a useable and accessible format?

Partly

Are the protocols appropriate and is the work technically sound?

Yes

Reviewer Expertise:

Mollusca, marine macroinvertebrates, genetics, genomics, biodiversity, evolution, phylogenetic studies, barcoding.

I confirm that I have read this submission and believe that I have an appropriate level of expertise to confirm that it is of an acceptable scientific standard, however I have significant reservations, as outlined above.

## References

[1] : A tale of two paths: The evolution of mitochondrial recombination in bivalves with doubly uniparental inheritance. *J Hered* .2023;114(3) : 10.1093/jhered/esad004 199-206 10.1093/jhered/esad004 36897956PMC10212130
